# Management and treatment of HIV: are primary care clinicians prepared for their new role?

**DOI:** 10.1186/s12875-020-01198-7

**Published:** 2020-07-01

**Authors:** Sampath Wijesinghe, Jeffrey L. Alexander

**Affiliations:** 1 College of Graduate Health Studies, A. T. Still University, 5850 East Still Circle, Mesa, AZ 85206 USA; 2 MSPA Education, Stanford School of Medicine, 1265 Welch Rd., Ste. 100, Stanford, CA 94305-5408 USA

**Keywords:** HIV, Primary care, Primary care providers, HIV provider shortage, Primary care workforce shortage, Challenges with HIV disease

## Abstract

**Background:**

Current literature suggests the number of HIV clinicians in the United States is diminishing. There are 294,834 primary care providers (PCP) in the United States, and, of these, 3101 provide care to HIV-positive patients. More PCPs to treat and manage HIV patients may be the solution to alleviate the HIV provider shortage. However, PCPs also face challenges, including workforce shortages. We surveyed PCPs to determine perceived barriers, beliefs, and attitudes about their readiness to manage and treat HIV patients.

**Methods:**

Following a quantitative, descriptive, cross-sectional survey design, currently practicing clinicians in primary care (physicians, residents, physician assistants, family nurse practitioners) were emailed a link to the study survey. Three hundred forty-seven family medicine clinicians from 47 states met the study inclusion criteria.

**Results:**

Most (245/347, 70.6%) of the PCPs agreed that PCPs should take care of HIV patients. PCPs practicing HIV medicine (*n* = 171) were more likely than those not practicing HIV medicine (*n* = 176) to agree that PCPs should help with the HIV provider shortage (*U* = 10,384, *p* < 0.001) and that PCPs are the best solution to the HIV provider shortage (*U* = 10,294, *p* < 0.001). The majority (206, 59.4%) believed PCPs are the best solution for the HIV provider shortage. Of 133 physician assistants (PAs) and family nurse practitioners (NPs), seventy (52.6%) believed they could be ready to manage HIV patients with some training.

**Conclusion:**

The HIV provider shortage in the United States is likely to continue. To alleviate the provider shortage, PCPs should be offered additional training, decreased workload, and increased compensation when treating and managing HIV patients. Also, encouraging PAs and family NPs to be involved with HIV medicine may be a solution.

## Background

Nearly 1.1 million persons in the United States (US) are living with HIV, and approximately 14% of them are unaware of their HIV-positive status [1]. The number of new HIV diagnoses annually has remained stable; 37,832 new HIV cases were diagnosed in 2018 [1]. While African-American homosexual men had the highest number of newly-diagnosed cases in 2018, a substantial increase was noted in Hispanic/Latino gay or bisexual men [1]. Overall, there have been 650,000 deaths from the AIDS epidemic [2]. Antiretroviral therapy (ART) is frequently recommended for HIV patients, because it stabilizes their immunity and helps them live longer and healthier lives [3]. However, ART is not a cure [3]. HIV-positive patients will continue to need sufficient number of HIV providers as the number of new infections continues and others who were previously diagnosed live longer.

According to the American Academy of HIV Medicine (AAHIVM) [4], over 32% of existing HIV clinicians will stop practicing HIV medicine over the next 10 years. More recently, Sweet [5] suggested that approximately 50% of HIV clinicians could retire in the next 5–10 years. Gatty [6] suggested that the reduction of HIV clinicians has already begun. A national survey of program directors of the Ryan White HIV/AIDS Program indicated a lack of qualified clinicians and poor reimbursement for HIV care are the primary challenges for recruitment of HIV clinicians [7]. The Mathematica Policy Research organization investigated the HIV provider shortage in 2016 and concluded that provider shortage increased from 7 to 30% between 2010 and 2015 [8]. The organization further concluded “we have a reason to believe this trend is continuing and will likely have long-term adverse consequences for public health if not addressed” [8]. While the number of HIV providers is decreasing, the number of individuals with HIV is increasing because of new HIV infections and greater HIV survival rates due to advanced ART [9]; therefore, it appears that primary care providers (PCPs) may need to help care for people living with HIV.

Research suggests the best approach to manage HIV patients involves a multidisciplinary team of HIV specialists, primary care physicians, family nurse practitioners (NPs), and physician assistants (PAs) [10]. Family NPs and PAs are useful additions to the team, because they combine quality HIV care with cost-effectiveness [11]. Researchers [12, 13] have found that teams of physicians, family NPs, and PAs achieve similar outcomes for HIV patients compared with physicians alone. Consequently, these teams of physicians, family NPs, and PAs could function as PCPs that effectively administer collaborative, interprofessional care for HIV patients.

Although primary care physicians can and do treat HIV patients [14], research findings suggest the primary care workforce will also experience a shortage of providers [15]. Other barriers that may keep PCPs from initiating HIV management and treatment are payment for medical services, government mandates, time, staffing and training, and work-life balance [16]. Even though the increased engagement of PCPs in the management and treatment of HIV patients may improve overall access to and quality of HIV care [17], little research has investigated whether PCPs are willing and prepared to manage and treat HIV patients. Therefore, the current study surveyed PCPs to determine perceived knowledge, beliefs, and attitudes about their readiness to manage and treat HIV patients.

## Methods

The current study used an anonymous, quantitative, descriptive, cross-sectional survey design. Convenience sampling was used to recruit potential participants. Study participants included US clinicians in primary care (physician, resident, PA, or family NP) currently practicing in a part-time or full-time capacity. Participants were located through primary care state and national associations, AAHIVM, universities, health systems, and social media networks (LinkedIn and Facebook). Clinicians who practiced in other fields (internal medicine, gynecology, sole pediatrics, or other) and students were excluded from participation. The local institutional review board considered the current study exempt from review.

### Survey development

Existing standardized survey instruments did not meet our preferred criteria for the current study; therefore, an online survey was created using SurveyMonkey (San Mateo, CA) (Appendix). The survey was developed specifically to evaluate the perceptions of PCPs regarding their knowledge, beliefs, and attitudes about their readiness to manage and treat HIV patients. Face and content validity were established through collaboration between the primary researcher and colleagues in family medicine. The colleagues evaluated the survey questions and provided constructive feedback to improve the validity. The survey was revised, and then sent to three family medicine clinicians to re-evaluate its content validity. With their additional feedback, the final survey was designed.

The survey included 36 closed-ended items and four key sections: demographics, knowledge, beliefs, and attitudes (Appendix). The demographics section included 15 items related to profession designation, area of practice, number of weekly hours in primary care, gender, age, number of years in practice, plans to retire, race/ethnicity, state of practice, practice location, practice setting, number of patients treated per day, if currently treating HIV patients in practice, number of HIV patients treated in practice (if applicable), and if the participant was a certified HIV specialist. Knowledge items used a three-point, Likert-like scale (yes, no, uncertain). Beliefs and attitudes items used a five-point Likert scale (strongly agree, agree, uncertain, disagree, and strongly disagree). An area for written comments was also provided at the end of the survey. The survey took about 10 min to complete.

### Data collection

Three emails were sent during the study. An initial email was sent to all potential study participants indicating they would be receiving the survey. At the same time, other entities (i.e., family medicine residency programs and health care systems) were contacted by email to ask for their help in reaching out to potential study participants. An email invitation with a link to the online survey was sent. By clicking on the link, participants were informed that they were providing consent to participate in the study. After 6 weeks, an email reminder was distributed. The survey was open for 3 months. As an incentive to participate, a raffle for a $25 Starbucks gift card was included at the end of the survey. Participants provided their email address to enter the raffle and were informed their email address would not be linked to their survey responses.

### Statistical analysis

Data collected were analyzed using IBM SPSS version 24.0 (Armonk, NY: IBM Corp.) statistical software. Incomplete surveys were excluded from analysis. Descriptive statistics were calculated for all variables using frequencies and percentages for categorical data and means and standard deviations (SDs) for continuous data. The Likert scale items were converted to a 3-point response scale (agree, uncertain, or disagree) prior to analysis. Not applicable answer choices in the survey were grouped under the uncertain category. Summary statistics were also calculated for subgroups of PCPs. Specifically, we summarized survey responses for beliefs and attitudes for all PCPs, only PCPs who indicated they were involved with HIV medicine, only PCPs who indicated they were not involved with HIV medicine, and only PAs and family NPs. The Kolmogorov-Smirnov Test of Normality was used to examine the distribution of the data, which revealed the data were not normally distributed (*p* < .05). Mann-Whitney U analysis was used to compare two survey items between PCPs who reported currently treating HIV patients and those who reported not currently treating HIV patients. The two survey items for comparison were related to whether PCPs should take care of HIV patients and if PCPs are the best solution to the HIV provider shortage. Significance was set a priori at *p* < .05, two-tailed.

## Results

Three hundred eighty-five PCPs from 47 US states responded to the survey. Of those, 347 surveys were included in the analyses; 38 surveys were excluded, because seven were incomplete, four were completed by PCPs in unrelated careers, and 27 were from different specialties.

### Demographic characteristics

Most respondents were female (202, 58.5%) and White (203, 58.5%) (Table 1). The mean (SD) age was 43·2 (11.9) years, mean number of hours worked weekly was 39.3 (15.5) hours, and mean number of years in practice was 12.2 (10.8) years. Most respondents (172, 49.6%) were physicians who worked fulltime (258, 74.4%). Two hundred ninety-nine (86.2%) consulted 10 patients or more in a day. Most (160, 46.1%) anticipated working more than 10 years, and most (101, 29.1%) resided in California. Diverse practice locations and practice settings were reported. One hundred seventy-one (49.3%) reported currently treating HIV patients.

**Table 1:** Demographic Characteristics of Primary Care Clinicians Who Completed the Survey (*N* = 347)

Demographic Characteristic	No (%) or Mean (SD)
Designation	
Physician	172 (49.6)
Resident physician	42 (12.1)
Physician assistant	67 (19.3)
Nurse practitioner	66 (19.0)
Extent of practice	
Full-time	258 (74.4)
Part-time	54 (15.6)
Locum/per diem	12 (3.5)
Other	23 (6.6)
Gender	
Male	142 (40.9)
Female	202 (58.5)
Transgender	2 (0.6)
Mean age	43·2 (11.9)
Mean number of hours weekly	39·3 (15.5)
Mean number of years in practice	12·2 (10.8)
Plan to retire	
Within 2 years	8 (2.3)
Within 2–5 years	16 (4.6)
Within 5–10 years	54 (15.6)
More than 10 years	160 (46.1)
No plan to retire	109 (31.4)
Race/Ethnicity	
White	203 (58.5)
Hispanic and Latino	42 (12.1)
Black or African American	25 (7.2)
Asian	42 (12.1)
Native Hawaiian and Other Pacific Islander	1 (0.3)
Other	34 (9.8)
Practice state	
California	101 (29.1)
Colorado	22 (6.3)
Florida	16 (4.6)
Illinois	14 (4.0)
Nebraska	15 (4.3)
New York	20 (5.8)
North Carolina	10 (2.9)
Pennsylvania	14 (4.0)
Texas	17 (4.9)
Washington	11 (3.2)
Remaining 37 states	107 (30.9)
Practice location	
Urban	167 (48.1)
Suburban	91 (26.2)
Rural	89 (25.7)
Practice setting	
Hospital	39 (11.2)
Solo practice	34 (9.8)
Group practice	112 (32.3)
Community care (rural health/federally qualified)	130 (37.5)
Other	32 (9.2)
Average number of patients treated in a day	
9 or fewer	48 (13.8)
10–19	180 (51.9)
20–29	104 (30.0)
More than 30	15 (4.3)
Currently treat HIV patients	
Yes	171 (49.3)
No	176 (50.7)
Certified HIV specialist	
Yes	80 (23.0)
No	224 (64.6)
No, but considering becoming an HIV specialist	42 (12.1)
Yes, but considering quitting the specialty	1 (0.3)

### Knowledge

Most PCPs had adequate knowledge about the management and treatment of HIV patients (Table 2). Although the majority (186, 53.6%) knew the number of new HIV diagnosis every year, 161 (46.4%) did not know or were uncertain.

**Table 2:** Primary Care Clinicians’ Knowledge About Management and Treatment of HIV

Survey Question	No. (%)		
	Yes	No	Uncertain
There is an HIV provider shortage in the United States	257 (74.1)	12 (3.5)	78 (22.5)
Because of antiretroviral therapy, HIV patients live longer now than in previous years	346 (99.7)	0	1 (0.3)
HIV is a chronic disease	340 (98.0)	5 (1.4)	2 (0.6)
Over the past 10 years, HIV treatments have advanced greatly	332 (95.7)	4 (1.2)	11 (3.2)
Every year there are about 50,000 new HIV patients	186 (53.6)	8 (2.3)	153 (44.1)

### Beliefs

Respondents’ beliefs are summarized in Table 3. Most PCPs (152, 43.8%) reported they did not have clinical knowledge to manage and treat HIV patients. Two-hundred seven (59.7%) were ready to take care of HIV patients with some training, and most (245, 70.6%) agreed PCPs should take care of HIV patients. When asked about compensation, 183 (52.7%) of PCPs were uncertain, and 138 (39.8%) believed HIV providers were not compensated sufficiently.

**Table 3:** Primary Care Clinicians’ Beliefs about Management and Treatment of HIV

Survey Question	No. (%)		
	Agree	Uncertain	Disagree
I have the necessary clinical knowledge to manage and treat HIV patients			
All PCPs	143 (41.2)	52 (15.0)	152 (43.8)
Only PCPs involved with HIV medicine	90 (70.2)	19 (11.1)	32 (18.7)
Only PCPs not involved with HIV medicine	23 (13.0)	33 (18.8)	120 (68.2)
Only PAs and NPs	44 (33.1)	19 (14.3)	70 (52.6)
I have the necessary education to manage and treat HIV patients			
All PCPs	161 (46.4)	48 (13.8)	138 (39.8)
Only PCPs involved with HIV medicine	125 (74.1)	15 (8.8)	31 (18.1)
Only PCPs not involved with HIV medicine	36 (20.4)	33 (18.8)	107 (60.8)
Only PAs and NPs	53 (39.8)	17 (12.8)	63 (47.4)
With some training, I will be ready to take care of HIV patients			
All PCPs	207 (59.7)	118 (34.0)	22 (6.3)
Only PCPs involved with HIV medicine	87 (50.8)	80 (46.8)	4 (2.4)
Only PCPs not involved with HIV medicine	120 (68.2)	38 (21.6)	18 (10.2)
Only PAs and NPs	70 (52.6)	55 (41.4)	8 (6.0)
Primary care clinicians should take care of HIV patients			
All PCPs	245 (70.6)	60 (17.3)	42 (12.1)
Only PCPs involved with HIV medicine	142 (83.0)	17 (9.9)	12 (7.0)
Only PCPs not involved with HIV medicine	103 (58.5)	43 (24.4)	30 (17.1)
Only PAs and NPs	70 (52.6)	37 (27.8)	26 (19.5)
The number of new HIV cases is stable in the United States; therefore, there is no need to worry about HIV anymore			
All PCPs	1 (0.3)	31 (8.9)	315 (90.8)
Only PCPs involved with HIV medicine	1 (0.6)	4 (2.3)	166 (97.0)
Only PCPs not involved with HIV medicine	0 (0.0)	27 (15.3)	149 (84.7)
Only PAs and NPs	0 (0.0)	13 (9.8)	120 (90.2)
Planning to resolve the HIV provider shortage is a priority of the healthcare agencies at this time			
All PCPs	109 (22.4)	119 (34.3)	119 (34.3)
Only PCPs involved with HIV medicine	57 (33.3)	45 (26.3)	69 (40.3)
Only PCPs not involved with HIV medicine	52 (29.5)	74 (42.0)	50 (28.5)
Only PAs and NPs	44 (33.1)	44 (33.1)	45 (33.8)
When treating HIV patients, clinicians are compensated sufficiently			
All PCPs	26 (7.5)	183 (52.7)	138 (39.8)
Only PCPs involved with HIV medicine	20 (11.7)	66 (38.6)	85 (49.7)
Only PCPs not involved with HIV medicine	6 (3.4)	117 (66.5)	53 (30.1)
Only PAs and NPs	7 (5.3)	77 (57.9)	49 (36.8)

*N* = 347 for all PCPs, *n* = 171 for only PCPs involved with HIV medicine, *n* = 176 for only PCPs not involved with HIV medicine, and *n* = 133 for only

PAs and NPs. Abbreviations: *NP* Nurse practitioner, *PA* Physician assistant, *PCP* Primary care provider (includes physicians, PAs, and NPs)

### Attitudes

Respondents’ attitudes are summarized in Table 4. More than half (193, 55.6%) of PCPs worried about the projected HIV workforce shortage. The majority (239, 68.9%) indicated they would like to treat HIV patients while providing primary care. Most (291, 83.8%) believed helping was important to alleviate the HIV provider shortage. One-hundred fifty-nine (45.9%) were not interested in attending salaried, HIV-specialist fellowship training, but over half (209, 60.2%) were interested in treating HIV patients if time permitted. Over half (179, 51.6%, *n* = 179) were also interested in treating HIV patients if they were compensated better, and most (206, 59.4%) believed PCPs were the best solution for the HIV provider shortage.

**Table 4:** Primary Care Clinicians’ Attitudes About Management and Treatment of HIV

Survey Question	No. (%)		
	Agree	Uncertain	Disagree
I worry about the projected HIV workforce shortage			
All PCPs	193 (55.6)	93 (26.8)	61 (17.6)
Only PCPs involved with HIV medicine	121 (70.8)	32 (18.7)	18 (10.5)
Only PCPs not involved with HIV medicine	72 (40.9)	61 (34.7)	43 (24.4)
Only PAs and NPs	67 (50.4)	41 (30.8)	25 (18.8)
There are enough other health care crises to worry about than HIV			
All PCPs	51 (14.7)	78 (22.5)	218 (62.8)
Only PCPs involved with HIV medicine	21 (12.3)	31 (18.1)	119 (69.5)
Only PCPs not involved with HIV medicine	30 (17.0)	47 (26.7)	99 (56.3)
Only PAs and NPs	15 (11.3)	31 (23.3)	87 (65.4)
I would like to take care of HIV patients while providing primary care			
All PCPs	239 (68.9)	68 (19.6)	40 (11.5)
Only PCPs involved with HIV medicine	155 (90.6)	11 (6.4)	5 (3.0)
Only PCPs not involved with HIV medicine	84 (47.7)	57 (32.4)	35 (19.9)
Only PAs and NPs	80 (60.2)	33 (24.8)	20 (15.0)
If a primary care clinician is the answer to alleviate HIV provider shortage, I should help out			
All PCPs	291 (83.8)	41 (11.8)	15 (4.4)
Only PCPs involved with HIV medicine	136 (97.0)	3 (1.8)	2 (1.2)
Only PCPs not involved with HIV medicine	125 (71.0)	38 (21.6)	13 (7.4)
Only PAs and NPs	107 (80.5)	20 (15.0)	6 (4.5)
I would like to attend 1–2 years HIV salaried specialist fellowship training, if available			
All PCPs	87 (25.1)	101 (29.1)	159 (45.8)
Only PCPs involved with HIV medicine	46 (26.9)	60 (35.1)	65 (35.0)
Only PCPs not involved with HIV medicine	41 (23.3)	41 (23.3)	94 (53.4)
Only PAs and NPs	47 (35.3)	39 (29.3)	47 (35.3)
I will consider taking care of HIV patients if I have enough time			
All PCPs	209 (60.2)	107 (30.9)	31 (8.9)
Only PCPs involved with HIV medicine	99 (57.8)	66 (38.6)	6 (3.5)
Only PCPs not involved with HIV medicine	110 (62.5)	41 (23.3)	25 (14.2)
Only PAs and NPs	77 (57.9)	44 (33.1)	12 (9.0)
I will consider taking care of HIV patients if I am compensated better			
All PCPs	179 (51.6)	92 (26.5)	76 (21.9)
Only PCPs involved with HIV medicine	95 (55.6)	35 (20.5)	41 (20.9)
Only PCPs not involved with HIV medicine	84 (47.7)	57 (32.4)	35 (19.9)
Only PAs and NPs	62 (46.6)	38 (28.6)	33 (24.8)
I am not interested in HIV medicine			
All PCPs	43 (12.4)	40 (11.5)	264 (76.1)
Only PCPs involved with HIV medicine	5 (3.0)	6 (3.5)	160 (93.6)
Only PCPs not involved with HIV medicine	38 (21.6)	34 (19.3)	104 (59.1)
Only PAs and NPs	20 (15.0)	19 (14.3)	94 (70.7)
Primary care providers are the best solution to the HIV provider shortage			
All PCPs	206 (59.3)	113 (32.6)	28 (8.1)
Only PCPs involved with HIV medicine	123 (71.9)	39 (22.8)	9 (5.3)
Only PCPs not involved with HIV medicine	83 (47.2)	74 (42.0)	19 (10.8)
Only PAs and NPs	65 (48.9)	50 (37.6)	18 (13.5)

*N* = 347 for all PCPs, *n* = 171 for only PCPs involved with HIV medicine, *n* = 176 for only PCPs not involved with HIV medicine, and *n* = 133 for only PAs and NPs

Abbreviations: *NP* Nurse practitioner, *PA* Physician assistant, *PCP* Primary care provider (includes physicians, PAs, and NPs)

### Responses from PCPs not involved with HIV medicine

Of the 347 study participants, 176 PCPs were not involved with HIV medicine. Of these, most (120, 68.2%) indicated they did not have the necessary clinical knowledge to manage and treat HIV patients; the same number believed they could be ready to manage and treat HIV patients with some training (Tables 3 and 4). The majority (103, 59.0%) agreed PCPs should manage and treat HIV patients, and most (125, 71.0%) believed helping was important to alleviate the HIV provider shortage. The majority (94, 53.4%) were not interested in attending salaried, HIV-specialist fellowship training but were interested in managing and treating HIV patients if time permitted (110, 62.5%). Almost half (84, 47.7%) were interested in treating HIV patients if they were better compensated (Table 4), and many (83, 47.2%) believed PCPs were the best solution for the HIV provider shortage.

### Responses from PAs and NPs

One hundred thirty-three PAs and NPs participated in the study. Seventy (52.6%) believed they could be ready to manage HIV patients with some training (Tables 3 and 4). Most (107, 80.5%) believed helping was important to alleviate the HIV provider shortage. An equal number of PAs and NPs (47, 35.3%) were interested or not interested in attending salaried, HIV-specialist fellowship training.

### Comparison analysis between groups

**Fig. 1:**
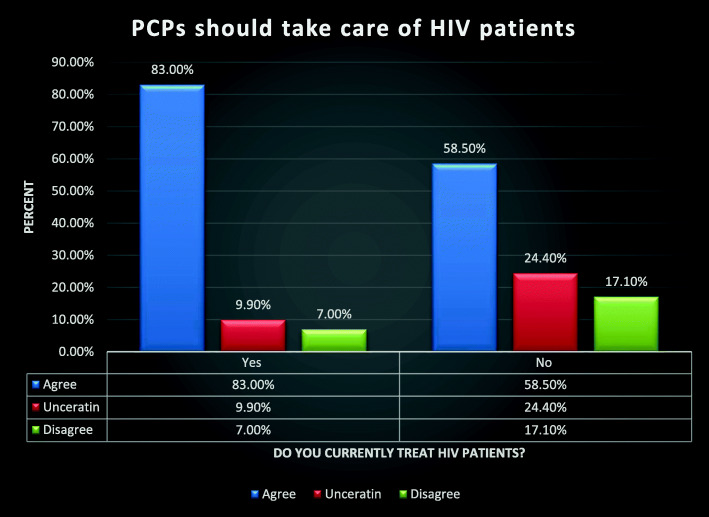
Comparison analysis between groups: PCP’s who currently practice HIV medicine vs. PCP’s who currently do not practice HIV medicine

**Fig. 2:**
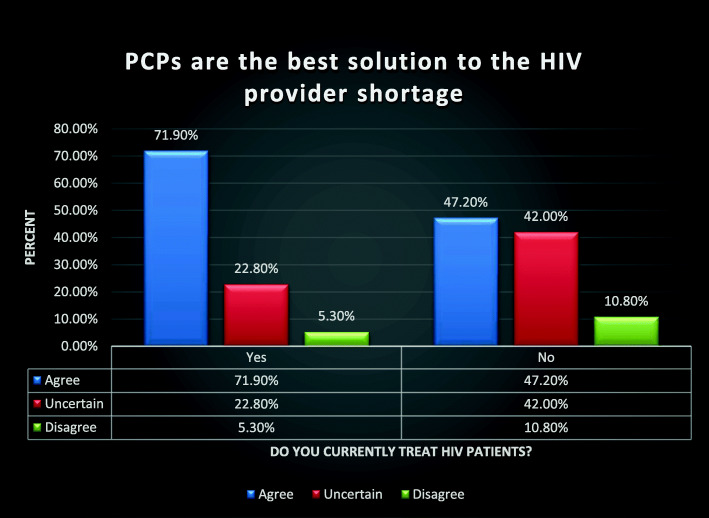
Comparison analysis between groups: PCP’s who currently practice HIV medicine vs. PCP’s who currently do not practice HIV medicine

One hundred seventy-one PCPs reported currently treating HIV patients, and 176 reported they did not. PCPs practicing HIV medicine were more likely than those not practicing HIV medicine to agree that PCPs should help with the HIV provider shortage (Fig. 1) (*U* = 10,384, *p* < 0.001) and that PCPs are the best solution to the HIV provider shortage (Fig. 2) (*U* = 10,294, *p* < 0.001).

## Discussion

We investigated whether PCPs were prepared to manage and treat patients with HIV. Overall, most respondents believed that PCPs should start managing and treating patients with HIV. Further, PCPs currently treating HIV patients were significantly more likely to agree that PCPs should take care of HIV patients than PCPs who were not.

There are 3101 PCPs in the United States who provide HIV care and consult with 10 or more HIV patients daily [18]. According to the Agency for Healthcare Research and Quality [19], there are currently 294,834 PCPs in the United States. Only a small percentage of PCPs manage and treat HIV patients; therefore, the responses of PCPs in the current study who did not treat and manage HIV patients are important, because they more representative of the overall primary care workforce.

For instance, our findings suggest PCPs should be more aware of some aspects of the HIV epidemic. Further, results suggest PCPs have concerns about provider salary, HIV provider training, and struggles with time limitations. Although PCPs had adequate knowledge about HIV and its basic elements, almost half did not know or were uncertain how many new HIV infections occur annually in the United States. Therefore, clinicians should be better educated about HIV to increase awareness of the problem.

In the current study, only 12% of PCPs treating HIV patients agreed they were compensated sufficiently for HIV care. This result suggested most PCPs involved with HIV medicine were unsatisfied with their salary. Moreover, 48% of PCPs who were not treating HIV patients indicated they would consider treating HIV patients if they were compensated better. Weddle and Hauschild’s  [7] findings support our finding that HIV clinicians are not compensated sufficiently. Traditionally, patients with HIV were treated and managed by infectious disease or HIV specialists. In contrast, when PCPs treat and manage these patients they manage, not only HIV, but also other chronic medical conditions; consequently, these patients receive comprehensive care, which can be complex and time consuming for providers. Taken together, our findings suggest PCPs expect higher compensation when managing HIV patients. Barakat [20] found patients are happy with this integrated model confirming they prefer this model, because they can receive comprehensive care (i.e., primary and HIV care) from one clinician [20]. This finding that patients with HIV prefer seeing PCPs, who provide primary and HIV care is important for PCPs as it may encourage them to consider getting more involved with HIV medicine.

Most PCPs in the current study indicated they lacked knowledge to treat and manage HIV patients, but most believed that they would be able to treat and manage HIV patients with some additional training. Weddle and Hauschild [7] found that PCPs were not qualified to manage and treat HIV patients. Gatty [6] proposed an HIV fellowship to increase the number of HIV providers. However, most PCPs in the current study did not like the idea of doing a lengthy HIV fellowship. As such, only a handful of US clinicians may be open to the idea of doing an HIV fellowship and becoming an HIV specialist. These results suggest PCPs may be interested in infrequent, but not long-term HIV-related training, such as HIV fellowships. The AIDS Education and Training Centers (AETC) are a network of eight regional training centers that provide HIV education [21]. Increasing the HIV workforce through proper training along the HIV care continuum is a priority for AETC [22]. Collaborating with the AETC to implement not only infrequent but also short-term HIV fellowships for PCPs may encourage them to attend HIV related training. Waldura [23] confirmed that PCPs who had access for HIV medical advice through a phone line (i.e., Warmline) reported that they are more confident with HIV care and are less dependent on referring patients to HIV specialists. These Warmline professionals are readily available for providers in regard to HIV related questions; particularly when dealing with complex cases. These phone consultations would also be useful for PCPs in rural locations. Considering the above findings, the AETC promotes more technological assistance to providers, who are involved with HIV medicine [22]. Apart from AETC, there are other clinicians’ Warmline available in the United States offered by other agencies like the Centers for Disease Control and Prevention (CDC) [24]. Therefore, educating PCPs about these resources and helpful services may encourage them to get involved with HIV medicine.

The Health Resources and Services Administration’s (HRSA) Ryan White HIV/AIDS program Part F Special Projects of National Significance (SPNS) Program supports the development of innovative models of HIV care and treatment, when necessary [25]. One of the SPNS initiatives includes system-level workforce capacity building for integrating HIV primary care in community health care settings. With this approach, the Program investigates how to respond to the changing health care landscape marked by shortages of HIV primary care physicians and increasing demand for access to quality HIV services [25]. Therefore, recognizing successfully implemented projects under SPNS and replicating them at more health care settings, and then educating clinicians about such available services may encourage them to become more involved with HIV medicine. According to HRSA’s Ryan White HIV/AIDS report, the Program served more than 551,000 people living with HIV – that is greater than half of all HIV patients in the United States [25]. The Program serves insured, uninsured, low-income, and underserved populations. Also, the Program covers medical services within HIV clinics and other health care settings. Patients are eligible for services provided by social workers, pharmacists, psychiatrists, and other providers. In 2016, about 63% of Program patients were living at or below 100% of the federal poverty level [25]. An opportunity to join an interdisciplinary team of providers through involvement with the Ryan White HIV/AIDS Program may motivate PCPs to consider engaging in HIV medicine. For example, a PCP can refer patients in need of behavioral health counseling to a psychiatrist and/or mental health counselor through the Ryan White HIV/AIDS Program, and all or most of the cost of these services would be covered [26]. Educating PCPs about these available services is vital to reduce perceived barriers towards and increase motivation for involvement in HIV medicine, thereby, increasing access to care for HIV infected patients.

Of PCPs not currently involved with HIV patients, 63% stated they would consider managing and treating HIV patients if time permitted. Bendix et al. [16] found that PCPs were burned out by their existing workload. Our findings support Bendix et al.’s, because our PCPs did not feel they had enough time. Overall, these finding suggest that a heavy workload is a barrier to different or additional responsibilities.

Research [12, 13] suggests that HIV clinician teams of PCPs achieve similar outcomes in the management of HIV patients when compared with physicians alone. In the current study, 81% of surveyed PAs and Family NPs believed they could help alleviate the HIV provider shortage by managing and treating these patients. Further, 35% were interested in attending salaried HIV specialist fellowship training. Given these results, a practical solution for addressing the HIV provider shortage may include reaching out to PAs and NPs and encouraging them to be involved with HIV medicine.

AIDS United is a nonprofit organization, which is strategizing to end the HIV epidemic (i.e., Getting to Zero) in the United States by 2025 [27]. Under the Getting to Zero initiative, the organization focuses on workforce recruitment and retention. AIDS united has halted funding elimination of SPNS and AETC in order to continue their efforts to alleviate the workforce provider shortage [28]. There are many resources available at these entities and programs (e.g., AIDS United, SPNS, AETC, CDC, and AAHIVM) for providers who are considering entering in to HIV medicine. Therefore, educating PCPs about such available services may encourage them to get involved with HIV medicine. To achieve Getting to Zero by 2025, the entities and aforementioned programs should continue their efforts to alleviate the HIV provider shortage.

The current study has some limitations. Although the percentage of PCPs in the United States practicing HIV medicine is small, many PCPs who were involved with HIV medicine completed our survey likely because of the survey distribution method. Therefore, our findings may not be representative of or generalizable to the overall population. To address this limitation in our analysis, responses from PCPs not involved with HIV medicine were compared against responses from all participants. Another limitation is the way the survey was distributed. One of our methods of distribution involved circulating the survey within US primary care residency programs. Some programs participated in the study and circulated the questionnaire within their systems. However, the number of surveys circulated in those systems is unknown, so a response rate could not be calculated. Participation bias may also be a limitation. Because completion of the survey was voluntary, only those PCPs already interested in HIV medicine may have responded. Another limitation was there was no survey question to identify if survey respondents were serving private or public insurance, which would have been an appropriate way to determine if PCPs were more involved with HIV care if patients had private versus public insurance due to different pay scales. Also, questions related to HIV-related stigma were not included. Geter et al. concluded “providers with limited recent HIV-stigma training were more likely to exhibit stigmatizing behaviors toward patients. Developing provider-centered stigma-reduction interventions may help advance national HIV prevention and care goals.” [29] A question about PCPs HIV-related stigma and their decision or willingness to care for and treat people living with HIV would have been useful for the current study. Further research is needed to determine if there is a correlation between PCPs HIV-related stigma and willingness to manage HIV infected patients, which understanding could help guide efforts to reduce barriers towards PCP involvement in HIV medicine. Additional research is also helpful to explore PCPs perceived knowledge, beliefs, and attitudes about their readiness to manage and treat HIV patients in states with high burden of HIV versus states with low burden of HIV in the United States. According to CDC, HIV diagnoses are unequally distributed regionally in the U.S. [1] In 2018, 15.7% cases were in the South; 12.7% were in the US dependent areas; 10% in the Northeast; 9.3% in the West; and 7.2% in the Midwest [1]. Such research will help determining if adequate number of PCPs are ready and/or willing to consider managing patients with HIV in those areas with higher prevalence.

While many PCPs of the current study were interested in managing and treating HIV patients, they faced critical barriers to doing so, such as lack of knowledge of HIV medicine, lack of time, and insufficient reimbursement. Further, although most PCPs understood the need for additional training to treat and manage HIV patients, our results suggested an HIV fellowship to provide training was not practical for most respondents. Therefore, short, regular training sessions to manage and treat HIV patients (i.e., a few hours weekly) may be the best solution, but these sessions should not impinge on routine clinical obligations. Another solution may be providing PCPs opportunities to participate in basic HIV training with the expectation that they will be able to manage and treat stable HIV patients while referring complex HIV patients to an HIV or infectious disease specialist. Because lack of time will continue to be a problem for PCPs, perhaps those who want to manage and treat HIV patients should have a lighter schedule, allowing them to give more time to HIV patients. Finally, steps should be taken to ensure that HIV providers are better compensated.

## Conclusion

In the current study, most PCPs agreed that they could be the solution to the HIV provider shortage and were interested in managing and treating HIV patients. However, our results suggest most PCPs in the United States are not involved with HIV medicine. Therefore, it seems the HIV provider shortage will continue. To alleviate this shortage and improve HIV patient care, PCPs should be offered additional training, decreased workloads, and increased compensation to better treat and manage HIV patients.

## Supplementary information

**Additional file 1: Appendix.** Survey Instrument.

## Data Availability

The dataset used and analyzed during the current study is available from the corresponding author on reasonable request.

## Abbreviations

PCP: Primary Care Provider
HIV: Human Immunodeficiency Virus
PA: Physician Assistant
NP: Nurse Practitioner
US: United States
AIDS: Acquired Immune Deficiency Syndrome
ART: Antiretroviral therapy
AAHIVM: American Academy of HIV Medicine
IBM: International Business Machines
SPSS: Statistical Package for the Social Science
SD: Standard Deviation
AETC: AIDS Education and Training Centers
CDC: Centers for Disease Control and Prevention
HRSA: Health Resources and Services Administration
SPNS: Special Projects of National Significance
CA: California
NY: New York
A.T.: Andrew Taylor (Acknowledgment)
UC: University of California (Acknowledgment)
PA-C: Physician Assistant Certified (Authors’ information)
