# Supplementary material for: Management and treatment of HIV: are primary care clinicians prepared for their new role?
Source: BMC Fam Pract. 2020 Jul 1;21:130. doi: 10.1186/s12875-020-01198-7 (PMC7330969; doi:10.1186/s12875-020-01198-7)
Supplement: Supplementary file 1 — Additional file 1: Appendix. Survey Instrument. [file 12875_2020_1198_MOESM1_ESM.docx]

Supplementary File

Survey Instrument

Management and Treatment of **HIV**: Are Primary Care Clinicians Prepared for the New Role?

**Demographics**.

1. I am a primary care

- Physician
- Resident Physician
- Physician Assistant (PA)
- Nurse Practitioner (FNP/DNP)
- Other ____________________

1. My area of practice

- Primary care/family medicine (full-time)
- Primary care/family medicine (part-time)
- Primary care/family medicine (Locum/ per-diem)
- Other _____________________

1. Number of weekly hours in primary care (rounded to the nearest whole number)

_________

1. Gender

- Male
- Female
- Transgender

1. My age

_________

1. Number of years in practice (rounded to the nearest whole number)

_________

1. I plan to retire

- Within 2 years
- Within 2-5 years
- Within 5-10 years
- More than 10 years
- No plan to retire

1. My ethnicity

- White American
- Hispanic and Latino American
- Black or African American
- Native American/Alaska Native
- Asian American
- Native Hawaiian and Other Pacific Islander
- Other _____________________

(H) My practicing state

- (i.e. Alabama) – pick your state from the list

1. My practice location

- Urban
- Suburban
- Rural

(J) My practice setting

- Hospital
- Solo practice
- Group practice
- Community care (Rural Health/ Federally Qualified)
- Other _________________________________________

(K) Average number of patients I treat in a day

- 9 or fewer patients
- 10- 19 patients
- 20- 29 patients
- More than 30 patients

(L) Do you currently treat HIV patients?

- Yes
- No

(M) If you see HIV patients, what is your HIV patient volume?

- Fewer than 19 patients
- 20-49 patients
- 50-99 patients
- More than 100 patients
- N/A (I do not treat HIV patients)

(N) I am a certified HIV specialist.

- Yes
- No
- No. But, I am considering becoming an HIV specialist
- Yes. But, I am considering to quit from my specialty

**Knowledge**

1. There is a HIV provider shortage in the United States *yes, no, uncertain*
2. Because of antiretroviral therapy, HIV patients live longer now than in previous years *yes, no, uncertain*
3. HIV is a chronic disease *yes, no, uncertain*
4. Over the past 10 years, HIV treatments have advanced greatly *yes, no, uncertain*
5. Every year there are about 50,000 new HIV patients *yes, no, uncertain*

**Beliefs**

1. I have the necessary clinical knowledge to manage and treat HIV patients *strongly agree, agree, uncertain , disagree, strongly disagree*
2. I have the necessary education to manage and treat HIV patients *strongly agree, agree, uncertain , disagree, strongly disagree*
3. With some training, I will be ready to take care of HIV patients *strongly agree, agree, uncertain , disagree, strongly disagree*
4. Primary care clinicians should take care of HIV patients *strongly agree, agree, uncertain , disagree, strongly disagree*
5. The number of new HIV cases is stable in the United States, therefore, there is no need to worry about HIV anymore *strongly agree, agree, uncertain , disagree, strongly disagree*
6. Planning to resolve the HIV provider shortage is a priority of the Healthcare agencies at this time *strongly agree, agree, uncertain , disagree, strongly disagree*
7. When treating HIV patients, clinicians are compensated sufficiently  *strongly agree, agree, uncertain , disagree, strongly disagree*

**Attitudes**

1. I worry about the projected HIV workforce shortage *strongly agree, agree, uncertain , disagree, strongly disagree*
2. There are enough other health care crises to worry about than HIV *strongly agree, agree, uncertain , disagree, strongly disagree*
3. I would like to take care of HIV patients while providing primary care *strongly agree, agree, uncertain , disagree, strongly disagree*
4. If a primary care clinician is the answer to alleviate HIV provider shortage, I should help out *strongly agree, agree, uncertain , disagree, strongly disagree*
5. I would like to attend 1-2 years HIV salaried specialist fellowship training, if available *strongly agree, agree, uncertain , disagree, strongly disagree*
6. I will consider taking care of HIV patients if I have enough time *strongly agree, agree, uncertain , disagree, strongly disagree*
7. I will consider taking care of HIV patients if I am compensated better *strongly agree, agree, uncertain , disagree, strongly disagree*
8. I am not interested in HIV medicine *strongly agree, agree, uncertain , disagree, strongly disagree*
9. Primary care providers are the best solution to the HIV provider shortage *strongly agree, agree, uncertain , disagree, strongly disagree*

If you have any comment regarding this subject, please write include your comments below:

*---------------------------------------------------------------------------------------------------------------------------------------------------------------------------------------------------------------------------------------------------------------------------------------------------------------------------------------------------------------------------------------------------------------------------------------------------------------------------------------------------------------------------------------------------------------------------------------------------------------------------------------------------------------------------------------------------*

**If you wish to be included for a raffle to win a $25 value Starbucks gift card please follow the directions after submitting your responses.**

| **Submit** |
| --- |

**Please enter your email below to be included for the raffle. Your email address will not be linked to survey responses.**

**-----------------------------------------------------------------------------**

| **Submit** |
| --- |
